# Caudal-dependent cell positioning directs morphogenesis of the *C. elegans* ventral epidermis

**DOI:** 10.1016/j.ydbio.2020.01.001

**Published:** 2020-05-01

**Authors:** Sophie P.R. Gilbert, Thomas W. Mullan, Richard J. Poole, Alison Woollard

**Affiliations:** a Department of Biochemistry, University of Oxford, South Parks Road, Oxford, OX1 3QU, UK; b Department of Cell and Developmental Biology, University College London, London, WC1E 6BT, UK

**Keywords:** *C. elegans*, Epidermis, Seam cells, Morphogenesis, *Caudal*, *Pal-1*

## Abstract

Strikingly, epithelial morphogenesis remains incomplete at the end of *C. elegans* embryonic development; newly hatched larvae undergo extensive remodelling of their ventral epidermis during the first larval stage (L1), when newly-born epidermal cells move ventrally to complete the epidermal syncytium. Prior to this remodelling, undivided lateral seam cells produce anterior adherens junction processes that are inherited by the anterior daughter cells following an asymmetric division during L1. These adherens junction processes provide the ventral migratory route for these anterior daughters. Here, we show that these processes are perturbed in *pal-1*/*caudal* mutant animals, resulting in their inheritance by posterior, seam-fated daughters. This causes aberrant migration of seam daughter cells, disrupting the ventral epidermis. Using 4D-lineaging, we demonstrate that this larval epidermal morphogenesis defect in *pal-1* mutants can be traced directly back to an initial cell positioning defect in the embryo. *pal-1* expression, driven by a single intronic enhancer, is required to correctly position the seam cells in embryos such that the appropriate cell junctions support the correct migratory paths of seam daughters later in development, irrespective of their fate. Thus, during ventral epithelial remodelling in *C. elegans*, we show that the position of migrating cells, specified by *pal-1/caudal*, appears to be more important than their fate in driving morphogenesis.

## Highlights

• *caudal*/*pal-1* is required to form the correct cell junctions during embryogenesis.
• Correctly placed cell junctions direct larval ventral epithelial cell migration.
• larval epithelial cell migration occurs independently of cell fate.
• Embryonic epidermal expression of *pal-1* is dependent on a single intronic enhancer.

## 1 Introduction

Morphogenesis is crucial to determine the final shape and function of tissues, organs and whole animals, yet the interplay between the various driving forces, such as cell migration and cell fate determination, is not well understood (Heisenberg and Solnica-Krezel, 2008; Chan et al., 2017). In *C. elegans,* as in most animals, the epidermis plays an instrumental role in coordinating morphogenesis. The adult epidermis is comprised, primarily, of a single multinucleate cell called the hyp7 syncytium that is first formed during embryogenesis. Two unfused rows of epidermal hyp7 cells intercalate with each other on the dorsal surface of embryos in a process known as dorsal intercalation (Sulston et al., 1983; Williams-Masson et al., 1998). This creates a single row of hyp7 that subsequently fuses to form the dorsal epidermis (Podbilewicz and White, 1994).

**Fig. 1:**
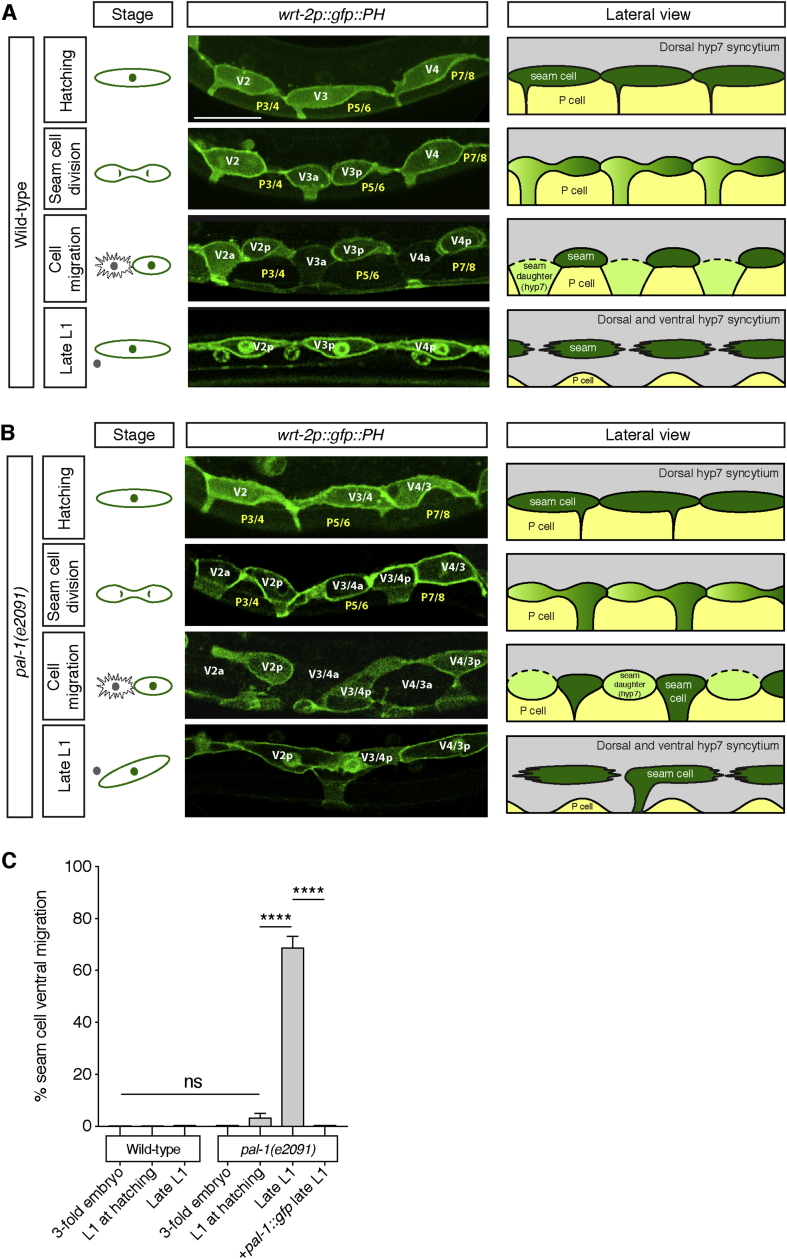
**Seam cells in *pal-1(e2091)* L1 larvae produce mis-polarised projections to the ventral midline causing incorrect seam daughter cell migration**. **A**. L1 seam cell division in wild-type animals, visualised using the seam expressed *wrt-2p*::*gfp*::*PH* (*heIs63*) reporter. For reference, stages of the asymmetric seam cell division are represented in the first column, showing cell division and subsequent hyp7 differentiation and fusion of the anterior seam cell daughter. Before division, the posterior seam cells V2-T possess an anteriorly polarised ventral projection that extends to the ventral midline between the P cells. As the seam cells divide, this projection widens as the P cells retract and migrate towards the ventral midline. At cytokinesis, this projection is inherited by the anterior, differentiating daughter cell, which loses *wrt-2* expression upon differentiation. The anterior daughter intercalates ventrally between the retreating P cells, before fusing to both neighbouring hyp7 daughters and the dorsal hyp7 by the end of L1. The final column shows a schematic of a lateral view of the L1 seam cell division and ventral intercalation of differentiating seam daughters with the P cells. The dorsal hyp7 is shown in grey, the seam cells in dark green and P cells in yellow. Seam daughters destined to differentiate to hyp7 are shown in light green. Solid black lines indicate apical junctions, whereas a dotted black line shows membranes undergoing cell fusion. B. L1 seam cell division in *pal-1(e2091)* mutant animals. Again, stages of cell division are represented in the first column. In *pal-1(e2091)* mutants, the projection from the L1 seam cells at hatching can be mis-polarised to the posterior, rather than anterior, end of the seam cell. As the cell divides, the posterior, seam-fated daughter inherits the projection erroneously and later moves out of the seam cell line to the ventral midline. Once the seam cells reconnect at their anterior and posterior ends at the end of L1, this results in a ventrally branched seam line. The identity of each seam cell (Vn, Vn+1…) is approximated with respect to its a-p position. As is evident in Fig. 5, embryonic lineaging later demonstrates that V3 and V4 become reversed in the seam line in *pal-1* embryos, thus V3 and V4 are likely to be also reversed at L1, hence the ambiguous labelling V3/V4. The last column shows a schematic of the ventral epidermis during the L1 seam cell division in *pal-1(e2091)* animals, with the same key as that in part A. C. Quantification of seam cell migration during early development in wild-type and *pal-1(e2091)* mutants [n ​> ​59 per strain per stage; ns P ​> ​0.05, ****P ​< ​0.0001, two-tailed Student’s t-test]. Seam cell branching occurs during L1 in *pal-1(e2091)* animals, but is never observed during equivalent stages in wild-type animals. A full-length genomic *pal-1*::*gfp* construct completely rescues the late L1 phenotype.

In contrast, the ventral epidermis undergoes extensive tissue remodelling during the first larval stage (L1), in which transiently-acting epidermal cells (P cells) are replaced by hyp7 syncytium. During the L1 stage, the P cells, which upon hatching make up the ventral surface of the worm, migrate to the ventral midline as they acquire neuroblast fate. At the same time, new hyp7 cells, born during the L1 stage from asymmetric divisions of lateral stem-like seam cells, move into the ventral epidermis (Sulston and Horvitz, 1977; Podbilewicz and White, 1994, see Fig. 1A and Supplementary Fig. 1). Importantly, only the hyp7-fated anterior daughters move ventrally as the P cells retreat, leaving their posterior seam-fated sister cells in their original lateral position. Thus, a line of lateral cells of alternating fates must coordinate so that only every other cell (the anterior hyp7 daughters) moves ventrally, whereas the cells between them (the posterior seam daughter cells) hold their lateral positions.

Previous observations have shown that undivided L1 seam cells extend distinct adherens junction processes from the anterior side of the cell towards the ventral midline. At division, therefore, these processes are always inherited by the anterior, hyp7-fated daughter, providing a migratory route for the cell towards the ventral midline to form the hyp7 ventral syncytium (Podbilewicz and White, 1994; Whangbo et al., 2000, see Fig. 1A). These polarised projections are unique to L1, and are not observed in seam cells later in post-embryonic development, during which they continue to divide in a stem-like manner (Sulston and Horvitz, 1977) without ventral processes.

Here, we investigate the role of the homeobox gene *pal-1/caudal* in regulating the polarisation of these ventral processes during epithelial morphogenesis, using a rare viable allele of *pal-1*, *e2091*. In these *pal-1* mutant animals, adherens junctions of a subset of seam cells are mispositioned in the newly hatched animal. Consequently, ventral migration is wrongly associated with the seam-fated (posterior) seam daughter, leaving the epidermally-fated (anterior) sister in the lateral position, thus perturbing morphogenesis. Furthermore, we demonstrate, using 4D-lineaging, that *pal-1* expression, driven by a single intronic enhancer in the same subset of epithelial cells, is required to correctly position the lateral seam cells with respect to the ventral P cells in embryos. This *pal-1*-mediated embryonic cell sorting positions adherens junctions to support the L1 ventral intercalation of only the anterior hyp7 epidermal daughters.

Overall, therefore, we have identified a novel role for *pal-1* in controlling embryonic epithelial cell movements. Loss of *pal-1* results in a disordered epithelium at hatching, which in turn affects a morphogenesis event later in development, during larval epithelial remodelling. We suggest that *pal-1-*mediated cell positioning is the crucial factor for driving the correct tissue remodelling during larval epithelial morphogenesis, irrespective of any overt changes in cell fate. The involvement of *pal-1* in regulating cell movement has resonance with mammalian studies, where *caudal* homologues are associated with tumour metastasis involving aberrant cell migrations (Mallo et al., 1998; Chun et al., 2007; Gross et al., 2008; Li et al., 2015).

## 2 Results

### 2.1 Adherens junction processes are mis-polarised in *pal-1* mutants, causing abnormal migration of seam cells

In wild-type, L1 seam cells produce an anteriorly polarised adherens junction process that ensures only the anterior seam daughter moves ventrally to form the ventral hyp7 syncytium (Fig. 1A). In order to investigate the mechanism controlling this polarity, we looked for mutants in which it was disrupted. In animals containing the viable *e2091* allele of the homeobox *caudal* transcription factor *pal-1*, we found abnormal posterior displacement of these processes in a subset of mid-body seam cells (Fig. 1B). As the L1 seam cells divide, the mis-positioned projection is erroneously inherited by the posterior, seam-fated daughter cell, causing this cell to move ventrally. These mispositioned posterior seam daughters retain their seam cell fate and therefore do not fuse with the hyp7 syncytium (Fig. 1B). As the seam cells reconnect with each other at their anteroposterior poles after the division, the edge of the ventrally mis-positioned cell remains anchored in the ventral midline, thereby causing a branched seam phenotype (Fig. 1B).

We quantified the proportion of animals containing at least one ventrally migrated seam cell between the end of embryogenesis and late L1 (Fig. 1C). Prior to the L1 division, seam cells are not displaced from their normal lateral positions, whereas approximately 70% of animals display a ventrally displaced seam cell after the L1 division. This abnormality is completely rescued by transgenic expression of a wild-type *pal-1* construct (Fig. 1C).

### 2.2 Abnormal ventral migration of seam cells results in branched alae

**Fig. 2:**
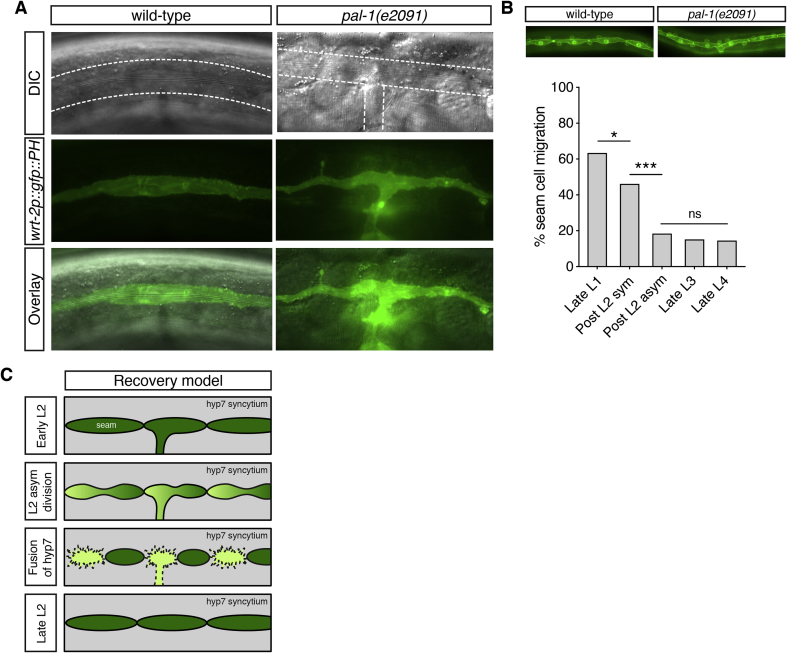
**Seam ventral branching in *pal-1(e2091)* adults causes abnormal alae, although seam morphology is often recovered during larval development**. **A**. Alae and seam branching phenotypes in *pal-1(e2091)* adults. 6% of animals at this stage (n ​= ​188) exhibit abnormal extensions of alae to the ventral midline in the mid region of the animal (alae highlighted between the dotted white lines), in each case corresponding to the shape of the underlying seam syncytium (GFP). B. Quantification of seam cell migration at each larval stage from late L1 to late L4. The two timings during the L2 molt make reference to the two seam divisions at that stage. Following L1, the seam cell branching phenotype drops in frequency within the population, with the greatest decrease observed following the L2 asymmetric seam cell division (‘Post L2 asym’) [n ​> ​50 per stage; *P ​< ​0.05, ***P ​< ​0.001, two-tailed Student’s t-test]. Seam branching is never observed in wild-type animals at any stage (data not shown). C. Model to explain the seam cell morphology recovery after an asymmetric seam cell division. As the branched seam cell divides asymmetrically, the ventral branch may be inherited by the anterior, differentiating seam daughter. Upon fusion to the hyp7, the abnormal branch of the seam line is lost. The hyp7 is shown in grey, the seam cells in dark green and seam daughters destined to differentiate to hyp7 are light green. Solid black lines indicate apical junctions, whereas a dotted black line shows membranes undergoing cell fusion. At these later larval stages, differentiating hyp7 daughters possess a ruffled membrane as they extend filopodia into the surrounding hyp7, thus migrating either dorsally or ventrally out of the seam line.

A small number of *pal-1(e2091)* adults exhibited abnormally shaped alae (longitudinal cuticular structures that are secreted by the adult, fully differentiated seam). Rather than the wild-type single lateral line of three cuticular ridges, 6% of *pal-1* mutant adults showed branched, ‘T-shaped’ alae (Fig. 2A). The branched alae extended towards the ventral midline from the mid-body region for each animal observed, consistent with the ventral branching of seam cells observed by 70% of animals at L1. We looked to see whether the shape of the fully differentiated seam syncytium was also branched in these animals. We found that, invariably, the pattern of the seam cell syncytium matched that of the branched alae (Fig. 2A); the exact correlation between seam position (even when aberrant) and the position of the alae provides evidence that alae production is autonomous and local to the seam cells. Animals with a branched seam syncytium were also phenotypically Egl (egg-laying defective, data not shown), presumably due to the disrupted ventral epidermis in the region in which the vulva forms.

The low penetrance of the T-shaped alae in adults may be explained by the recovery of normal seam cell morphology through the continued asymmetric division of the branched seam cell after L1 and subsequent fusion of anterior daughters to the hyp7. This fusion would lead to loss of attachment to the ventral midline and thereby reestablish normal seam cell contacts in many cases as larval development progresses. Recovery would also be consistent with the absence of larval lethality we observed in *pal-1(e2091)* animals (n ​= ​1742). In order to observe recovery of seam cell morphology, we quantified seam cell ventral branching from late L1 to late L4 (Fig. 2B). These data show that the incidence of ventral seam cell displacement decreases throughout larval development, with the greatest recovery occurring after the L2 asymmetrical division that generates a hyp7 daughter (Fig. 2B, ‘Post L2 asym’). Presumably, neither seam-fated daughters of the preceding L2 symmetric division (‘Post L2 sym’) can fuse to hyp7 and resolve the branch (Fig. 2C). The small decrease in the incidence of ventral displacement between late L1 and the L2 symmetric division is likely due to the fragility of the epidermis in *pal-1(e2091)* mutants at this stage that results in rupture during slide preparation. By adulthood, therefore, the incidence of seam cell ventral displacement is low.

This recovery phenotype suggests that displaced seam cells in *pal-1(e2091)* mutants retain their seam identity of further proliferation. The developmental fate of ventrally migrated seam cells in *pal-1(e2091)* mutants therefore appears robust to changes in their position, as evidenced by their continued ability to divide, lack of fusion to hyp7, eventual production of alae and continued expression of *wrt-2*.

### 2.3 Developmental basis of mis-polarisation of seam daughter cell projections in *pal-1(e2091)*

**Fig. 3:**
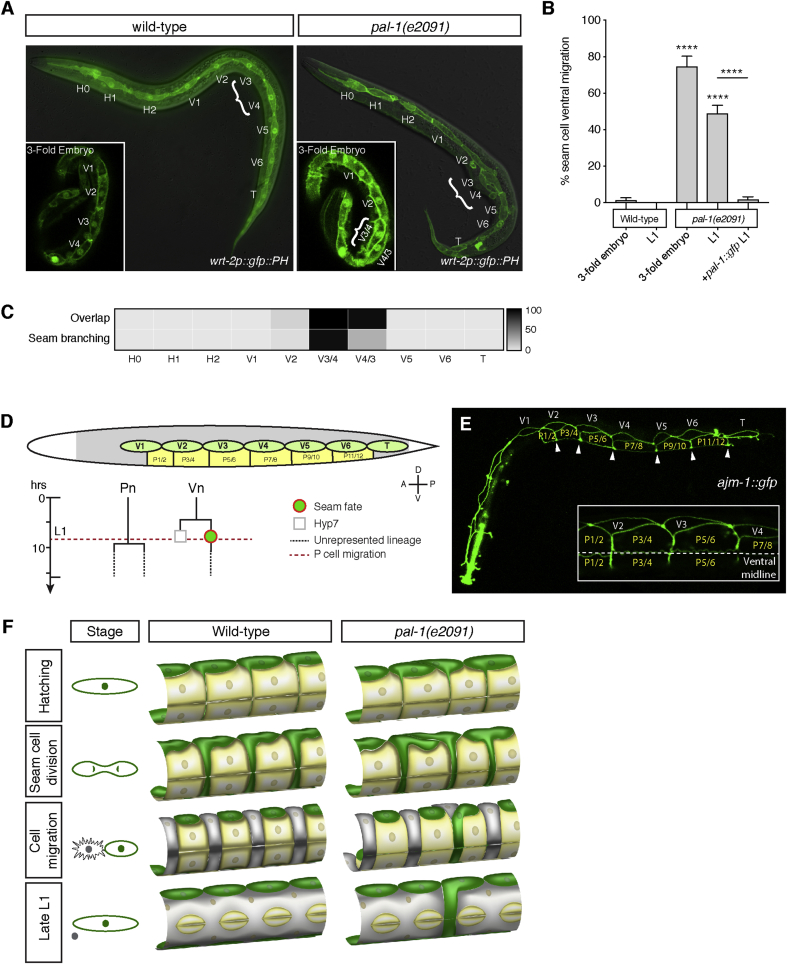
**Mis-polarisation of *pal-1(e2091)* seam cell processes at L1 results from mis-alignment of epidermal cells at hatching.** **A**. Seam cells in a wild-type and *pal-1(e2091)* mutant at the end of L1. *pal-1(e2091)* animals exhibit an abnormal clustering of seam cells in the mid-body region, with V lineage cells sharing anteroposterior axes (bracket). This ‘overlap’ phenotype can already be observed in the late (3-fold) embryo (insets). **B.** Quantification of the seam cell overlap during early development in wild-type and *pal-1(e2091)* mutants [n ​> ​58 per strain per stage; ****P ​< ​0.0001, two-tailed Student’s t-test]. Overlapping seam cells can be observed as early as the 3-fold embryo in *pal-1(e2091)* animals. A full-length genomic *pal-1*::*gfp* construct completely rescues the L1 phenotype. **C.** Heat map showing the percentage of cells involved in either the ‘overlap’ or the ventral branching seam phenotype in *pal-1(e2091)* animals. The identity of each seam cell (Vn, Vn+1…) is approximated with respect to its a-p position. As is evident in Fig. 5, embryonic lineaging later demonstrates that V3 and V4 become reversed in the seam line in *pal-1* embryos, thus V3 and V4 are likely to be also reversed at L1, hence the ambiguous labelling V3/V4. There is a strong correlation between cells that are involved in the seam cell overlap phenotype and the identities of those seam cells that migrate to the ventral mid-line [two-way anova, P ​< ​0.0001]. **D.** Schematic showing the position of medial and posterior seam (Vn and T) and P cells in a wild-type worm at hatching. The seam cells (green) lie in two lateral rows either side of the animal, and at this stage the P cells (yellow) lie ventrally. The dorsal hyp7 syncytial epidermis is shown in grey. L1 lineages of the Pn and Vn cells are represented along with a dashed red line indicating the time in L1 at which the P cells migrate to the ventral midline (timings from Sulston and Horvitz, 1977). **E.** Seam and P cells at hatching, showing the apical junctions between cells using *ajm-1*::*gfp*. Arrowheads indicate the apical junctions between P cells where the seam cell projection forms during division. The inset shows a lateral-ventral view of P cells either side of the ventral midline. **F.** Graphic representation of the lateral-ventral surface in wild-type and *pal-1(e2091)* animals during L1. The first column indicates the stage of seam cell division. In wild-type, seam cells are lined up with respect to the P cells such that their anterior portion is adjacent to the apical junction between P cells. Seam cells form a process to the ventral midline through this junction. During division, the P cells shrink and migrate to the ventral midline, allowing the seam cell anterior process to form a pathway through which the anterior seam cell daughter can migrate into the ventral space. Posterior seam-fated daughters retain their lateral position. *pal-1(e2091)* mutant animals hatch with a mis-alignment of seam cells in the mid-body region, the ‘overlap’ phenotype. This causes seam cells in this region to be mis-aligned with respect to the ventral P cells, such that the apical junction between P cells in this region lies adjacent to the posterior, not anterior, portion of the seam cell. This seam cell, therefore, extends a process from the posterior side of the cell to the ventral midline, through the P cell apical junction. As seam cell division progresses, the wrong cell, *i.e.* the posterior seam cell daughter, inherits the process and erroneously moves into the ventral space in place of the anterior hyp7 daughter. This misplaced cell retains the seam cell proliferative fate and does not fuse to hyp7.

*pal-1(e2091)* L1 larvae have been shown to have a characteristic ‘seam cell overlap’ phenotype (Edgar et al., 2001, Fig. 3A), which we speculated could be the phenotypic origin of the mis-polarised seam cell adherens junction projections at L1. By inspecting embryos, we observed the same ‘overlap’ phenotype at the 3-fold stage, in which a sub-set of mid-body seam cells are wrongly positioned (Fig. 3A). We quantified the seam cell overlap phenotype in *pal-1(e2091)* animals, finding that the incidence of seam disorganisation is around 70% during late embryogenesis and upon hatching (Fig. 3B), similar to the penetrance of the mis-polarised projection phenotype. The high incidence of seam cell overlap in L1 is completely rescued by a full-length *pal-1*::*gfp* transgene (Fig. 3B).

In order to establish the precise relationship between the seam cell overlap phenotype and the aberrant seam cell migration, we quantified both phenotypes for each seam cell along the anteroposterior axis (H0-T, assigned by their anteroposterior position relative to each other), finding that mid-body seam cells V3 and V4 are the most commonly affected for both phenotypes (Fig. 3C). Thus, although the seam cell overlap phenotype has an origin much earlier during development, there is a very tight association between the cells involved in the overlap and those that subsequently migrate aberrantly during L1.

In the mid-body of wild-type worms, the anterior portion of each seam cell lines up with the junction between adjacent ventral P cells (Fig. 3D–E). The ventral projections from dividing seam cells follow these junctions (presumably the “path of least resistance”), such that, as the seam cell starts to divide, the widening projections are always inherited by the anterior daughter cell. This provides a ventral pathway through which the anterior daughter moves as the P cells migrate to the ventral midline (see Supplementary Fig. 1). Conversely, the posterior, seam-fated daughter cell loses the connection to the projection after cytokinesis, and thus retains its lateral position (Fig. 3F). In *pal-1(e2091)*, however, the seam cell overlap changes the positional relationship between the V and P cells such that the posterior, rather than the anterior, half of the seam cell now lines up with the junction between P cells (Fig. 3F). Ventral projections from these cells are, therefore, in position to be inherited erroneously by the posterior, seam-fated daughter, causing this cell to move ventrally and intercalate with the P cells.

The relative positioning of the seam cells with respect to the ventral P cells therefore appears to be the major determinant of correct seam daughter cell migration.

### 2.4 The formation of ventral projections is not dependent on cell fate

**Fig. 4:**
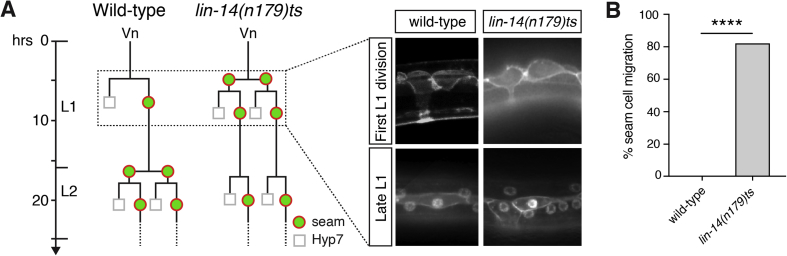
**hyp7 fate is not required for seam daughter cell ventral migration.** **A**. Seam V cell lineages of a wild-type and *lin-14(n179)ts* animal during L1 and L2. Seam cells in *lin-14(n179)ts* animals grown at the restrictive temperature divide symmetrically to the seam cell fate at the start of L1, precociously undergoing the wild-type L2 pattern of cell divisions during L1. Dotted lines indicate an unrepresented lineage. As in wild-type, the anterior daughter of the first L1 division in *lin-14* animals extends a process to the ventral midline, despite being of seam cell fate rather than hyp7 fate. By late L1, this *lin-14* anterior seam daughter has moved ventrally and forms a branched seam cell morphology. **B.** Inappropriate seam cell migration to the ventral midline at the end of L1 in wild-type and *lin-14* animals grown at the restrictive temperature [n ​> ​61 per strain; ****P ​< ​0.0001, two-tailed Student’s t-test].

We have shown how the mis-positioning of the seam cells with respect to the P cells in the epidermis is enough to switch morphogenetic movement from a hyp7-fated cell to a seam-fated cell. But does switching cell fate impact morphogenesis? To test this, we used a mutant of the heterochronic gene *lin-14* to perturb seam daughter cell fate after division in L1. In these animals, the first L1 division of seam cells is symmetrised, producing two seam-fated daughters, and therefore the hyp7 fate of the anterior daughter cell is lost (Ambros and Horvitz 1984, 1987) (Fig. 4A). We found that, despite the fate switch of the anterior daughter to the proliferative seam fate, the formation of a ventral projection from the anterior daughter cell still occurs (Fig. 4A). Quantifying this, we observed that by the end of L1 over 80% of animals exhibit seam cells embedded in the ventral midline (Fig. 4A–B). From this we can conclude that hyp7 fate is not required for epidermal cell ventral movement during L1 morphogenesis. Taken together, these data support the hypothesis that the arrangement of epithelial cell junctions positions the ventral projection during L1 morphogenesis, which in turn is sufficient to direct cell movement, irrespective of daughter cell fate.

### 2.5 *pal-1* is essential for correct epithelial cell sorting in the embryo

**Fig. 5:**
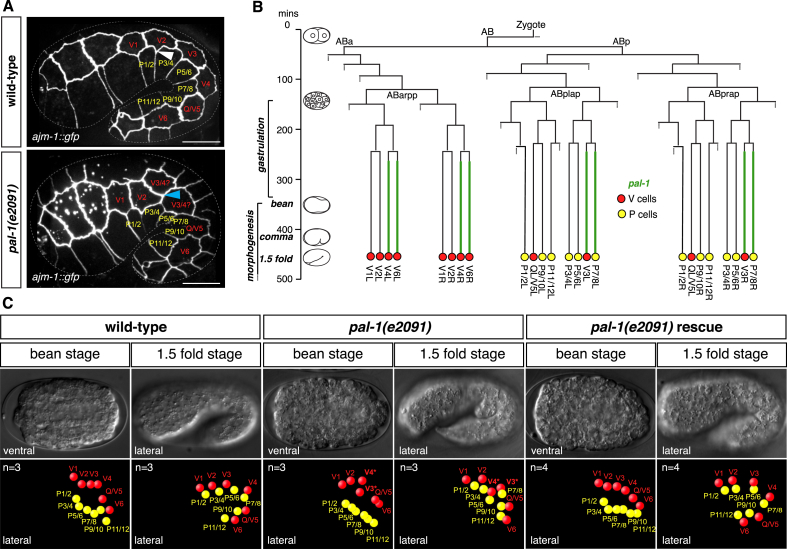
***pal-1* is required for seam organisation in the embryo.A**. Maximum intensity projections of lateral z stacks of wild-type and *pal-1(e2091)* 1.5 fold embryos carrying the *ajm-1*::*gfp* apical junction marker (strain AW298). The V lineage seam cells and the P cells are labelled. In wild-type embryos, the seam cells are ordered end to end along the a-p axis, and the apical junction between the ventral P cells lies towards the anterior of the ventral edge of each seam cell (example shown by the white arrowhead). In *pal-1(e2091)* animals the V3 and V4 seam cells are misaligned and partially share a-p coordinates. Misalignment of V3 and V4 skews the position of a ventrally joining apical junction such that it lies to the posterior (rather than the wild-type anterior) of the seam cell (blue arrowhead). Scale bar ​= ​10 ​ ​μm. **B.** Partial embryonic cell lineage of a wild-type embryo (re-drawn using timings from Sulston et al., 1983). Minutes from the first cleavage as well as timings of embryonic stages, gastrulation and morphogenesis are shown on the left. Green lines represent the expression of a rescuing full-length *pal-1*::*gfp* construct (n ​= ​4). Dotted lines represent additional unrepresented lineages. **C.** Embryonic 4D-lineaging of the V seam cells and P cells in wild-type animals (n ​= ​3 lineages), *pal-1(e2091)* mutants (n ​= ​3 lineages) and *pal-1(e2091)* carrying a full-length *pal-1*::*gfp* transgene (n ​= ​4 lineages) reveals the cell sorting defects of V3 and V4 in *pal-1* mutants that are fully rescued by the transgene. For each genotype a representative DIC image and 3D-position of the V and P cells in the embryo are shown for the bean and 1.5 fold stages. Asterisks indicate the cells which are abnormally positioned in the *pal-1(e2091)* embryo; by bean, V3 has failed to migrate to its proper position in the line of seam cells. Thus, as elongation of the embryo occurs and the animal reaches 1.5 fold, V3 remains to the posterior of V4 (therefore, from position alone, the identity of these cells cannot be determined, see Fig. 1, Fig. 3).

We have already found that the overlapping seam cell defect can be observed as early as the 3-fold embryo (Fig. 3A), suggesting that *pal-1* acts earlier in embryogenesis to specify relative epidermal cell positioning. Taking advantage of the fully determined, invariant lineage of *C. elegans*, we were able to precisely identify the embryonic origin of the epidermal patterning defect in *pal-1(e2091)* mutants. We carried out 4D-lineage analysis of V and P cells in both wild-type and *pal-1(e2091)* mutant embryos. The twelve V lineage seam cells and the twelve P cells are all born from the AB embryonic lineage (Sulston et al., 1983, Fig. 5B). All of the P cells originate from ABplap and ABprap lineages, and most of the V cells are born as pairs of sister cells in the ABarpp lineage. The exceptions to this are V3 and V5, each born from an asymmetric division in the ABp(l/r)ap lineages which also gives rise to P cells. V5 is the latest seam cell born, just prior to hatching, as a sister of the Q neuroblast, and V3 is born as the sister of P7/8 (Fig. 5B). This means that V3 and V5 have a lineage origin much more closely aligned to that of the P cells than the rest of the V cells. Thus, in wild-type animals, V3 is born in a cluster with the P cells and subsequently migrates into the line of V cells after they are born. This requires a precise intercalation of V3 between V2 and V4 by bean stage, in order to establish the correct anteroposterior order of cells in the seam line prior to embryonic elongation (Fig. 5C, Supplementary movie 1).

Supplementary video related to this article can be found at doi:10.1016/j.ydbio.2020.01.001.

The following is the supplementary data related to this article:

Supplementary movie 1 Lineaged wild-type embryo showing the birth of the P cells (yellow) and the seam cells (red) from the AB lineage. Cell positions are shown through to the 1.5 fold stage. Each ball represents a nucleus. Supplementary movie 1

As the wild-type animal begins to elongate along the anteroposterior axis and approaches the 1.5 fold stage, the single lateral line of seam cells lines up out of phase with the more ventral single line of P cells on each side of the animal, such that the cell contacts between P cells lie to the anterior end of each seam cell (Fig. 5A and C). It is this positioning of the junctions between P cells with respect to the seam cells in embryos that acts to position the adherens junction projections that develop in L1 to support correct cell migration.

In contrast, V3 in the *pal-1(e2091)* mutant, although born in the correct place, fails to move from the P cell cluster into the seam line by the bean stage (Fig. 5C, Supplementary movie 2). V4 now makes an aberrant contact with V2 in the absence of V3, closing the gap in the seam line, by which time V3 has been pushed more posteriorly and can no longer join the single line of lateral seam cells. Thus, by bean stage, the *pal-1(e2091)* animal has an abnormally patterned epithelium in which V3 lies posteriorly and dorsally to V4. As a result of this positioning defect, the adherens junction between the underlying P cells is now displaced from the anterior of V4 towards the posterior (Fig. 5A). Any projection that now forms ventrally from this cell through the widening of this junction during L1 is consequently posteriorly localised. Therefore, *pal-1* is required for the correct alignment of the seam and P cells shortly after they are born in the embryo, and this in turn influences the correct positioning of dividing seam cell projections in L1 larvae.

Supplementary video related to this article can be found at doi:10.1016/j.ydbio.2020.01.001.

The following is the supplementary data related to this article:

Supplementary movie 2 Lineaged *pal-1(e2091)* embryo showing the birth of the P (yellow) and seam cells (red) and positioning through to the 1.5 fold stage. Supplementary movie 2

The intercalation defect of V3 into the seam line in *pal-1(e2091)* mutants is completely rescued by expression of a full-length *pal-1*::*gfp* construct (Fig. 5C, Supplementary movie 3).

Supplementary video related to this article can be found at doi:10.1016/j.ydbio.2020.01.001.

The following is the supplementary data related to this article:

Supplementary movie 3 Lineaged embryo of genotype *pal-1(e2091); unc-119(ed3)* carrying the rescuing *pal-1*::*gfp* array. P cell nuclei are represented in yellow, seam cells in red. Supplementary movie 3

### 2.6 Embryonic *pal-1* expression in V and P cells is completely lost in the *e2091* allele

**Fig. 6:**
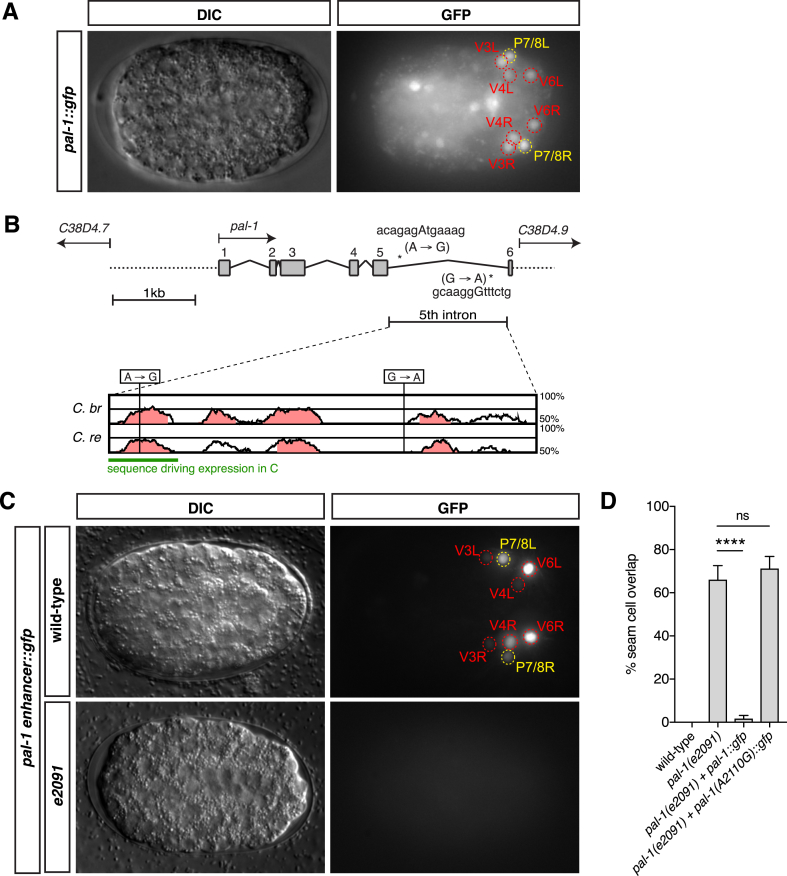
**Expression of *pal-1*::*gfp* in V seam and P cells is dependent on expression driven from a *pal-1* intronic enhancer, perturbed in *e2091***.**A**. Ventral view of embryos at dorsal intercalation carrying the wild-type rescuing full-length *pal-1*::*gfp* construct. Expression can be observed in multiple cells including V3, V4 and V6 and P7/8 (the duration of this expression is represented in Fig. 5B). B. The genomic structure of *pal-1*. Exons are represented as grey boxes, and the positions of the two point mutations in the last intron in *e2091* are indicated by asterisks. Conservation of the *pal-1* 5th intron, containing the two point mutations of *e2091*, between two members of the *Caenorhabditis* species, *C. briggsae* and *C. remanei* is shown below (generated using Frazer et al., 2004). A green stripe indicates the sequence cloned and used to assess enhancer function *in vivo*. C. Ventral view of embryos at dorsal intercalation carrying either the wild-type or *e2091* mutated version of a construct containing three copies of the first conserved non-coding element of the last *pal-1* intron (sequence indicated in B in green). The wild-type enhancer-only construct is able to drive GFP specifically in V3 and P7/8 sister nuclei, as well as the pair of sisters V4 and V6. Conversely, the construct carrying the *e2091* version of the putative enhancer is unable to drive GFP expression. The GFP images represent maximum intensity projections of a z stack through the whole embryo; DIC images comprise a single representative z slice that corresponds to cells expressing GFP. D. Rescue of *pal-1(e2091)* L1 seam cell phenotypes by *pal-1*::*gfp* is eliminated by the introduction of the *e2091* point mutation A2110G into the 5′ intronic enhancer [n ​> ​50, ****P ​< ​0.0001, two-tailed Student’s t-test].

We detected embryonic expression of our rescuing *pal-1*::*gfp* construct, containing the full genomic sequence of *pal-1*, in two pairs of epidermal daughter cells, V3 and P7/8, and V4 and V6, from shortly after their birth (Fig. 5, Fig. 6A). In contrast, we were unable to detect any expression of *pal-1* in L1 larvae, supporting the hypothesis that *pal-1* functions in embryos to coordinate L1 ventral epithelial morphogenesis. The *e2091* allele contains only two point mutations in the last intron of *pal-1* (Zhang and Emmons, 2000) (Fig. 6B), prompting intriguing questions about the possible role of this intron in directing *pal-1* expression in epidermal cells. The last intron of the *C. elegans pal-1* gene is remarkably well conserved with that of two other *Caenorhabditis* species, *C. briggsae and C. remanei* (Supplementary Fig. 2), with multiple highly conserved sequence domains (Fig. 6B). The 5′-most *e2091* point mutation (A2110G) lies in one of these conserved regions (Fig. 6B), and has previously been reported to be the phenotype-causing mutation in this allele, from a reversion event isolated in a suppressor screen for *pal-1(e2091)* male tail defects (Zhang and Emmons, 2000).

To test whether the last intron of *pal-1* contains an enhancer for embryonic V3 cell expression, we created two equivalent expression constructs and compared expression of these constructs to that of the full-length *pal-1*::*gfp* rescue construct. The first construct contained three wild-type copies of the 100bp region spanning the first conserved element, in which the A2110G *e2091* point mutation occurs, driving GFP expression (green bar, Fig. 6B). The second construct was identical to the first one, except that we introduced the A2110G *e2091* point mutation into the concatemerised region. We found that the former construct was able to drive GFP expression in only the two epidermal sister cell pairs, V3 and P7/8 and V4 and V6 (approximated by cell position in two out of two transgenic lines generated), whereas the latter construct was unable to drive any GFP expression (in two out of two transgenic lines generated) (Fig. 6C). Introducing the A2110G *e2091* mutation into the full length *pal-1*::*gfp* reporter was found to prevent rescue of the abnormal epidermal cell positions at hatching (Fig. 6D). Therefore, we conclude that the last intron of *pal-1* contains an enhancer that is necessary and sufficient to drive *pal-1* expression specifically in epidermal cells in order to regulate embryonic cell sorting and subsequent epidermal morphogenesis.

Using transcription factor binding site prediction software (Mathelier et al., 2014), we found that the region overlapping the A2110G mutation bears similarities to a TCF binding site, with no other obvious transcription factor binding sites in this region (Supplementary Fig. 2). This raised the possibility that *pal-1* acts directly downstream of Wnt signalling to arrange epidermal cells in the embryo, which would be intriguing in the light of the well-charcterised roles of Wnt signaling in controlling A-P polarity. Testing Wnt pathway mutants for phenocopy of *pal-1* is difficult because they are often lethal very early on in embryonic development (Lin et al., 1998), but by using a temperature-sensitive allele of *wrm-1*/β-catenin we were able to temperature shift animals partway through embryogenesis. We found that, by perturbing the Wnt pathway this way, we were able to observe a phenotype of overlapping seam cells at L1 (Supplementary Figs. 3A and B) without losing seam cell identity (Supplementary Fig. 3C). However, this overlap phenotype differs from the *pal-1* seam overlap phenotype in that it does not only involve the mis-positioning of V3/V4, but rather occurs anywhere in the V lineages of the seam (Supplementary Fig. 3A). Because of this, this newly identified phenotype of *wrm-1* is not sufficient evidence for a direct regulatory interaction between Wnt and *pal-1*.

## 3 Discussion

### 3.1 *pal-1(e2091)* is a strong loss-of-function mutant for embryonic V/P cell sorting

*e2091* is an unusual allele of *pal-1,* in that the animals are viable; null mutants of *pal-1* die during embryogenesis due to its requirement for ventral enclosure and fate patterning of posterior tissues (Hunter and Kenyon, 1996; Ahringer, 1997; Edgar et al., 2001; Baugh et al., 2005; Maduro et al., 2005; Lei et al., 2009). This is consistent with the role of *caudal* in other organisms (Macdonald and Struhl, 1986; Mlodzik et al., 1990; Epstein et al., 1997; Marom et al., 1997; Isaacs et al., 1998; Moreno and Morata, 1999; Ehrman and Yutzey, 2001; van den Akker et al., 2002; Chawengsaksophak et al., 2004; Copf et al., 2004; Shimizu et al., 2005; Shinmyo et al., 2005). It has previously been found that *e2091* affects the development of the male tail due to fate transformations in the male-specific seam lineage of the V6 seam cell (Waring and Kenyon, 1990) but its effect on hermaphrodite seam development had not been well studied. Importantly, although *e2091* animals are viable, it has been shown genetically, using a deficiency, that *e2091* is in fact a strong loss-of-function allele for the seam cell disorganisation (‘seam overlap’) phenotype of *pal-1* (Edgar et al., 2001). This, taken together with our finding that embryonic *pal-1* expression in V and P cells was completely abrogated by introduction of the *e2091* mutation, suggests that the embryonic V/P sorting defect is likely a specific, but complete loss-of-function phenotype of *pal-1* in the *e2091* allele.

The observed expression driven by the enhancer, perturbed by the *e2091* allele, is highly specific and likely required to ensure tight temporal and spatial control of *pal-1*. Given that the only other phenotype associated with the *e2091* allele is a defect in the post-embryonic V6 seam lineage of the male tail (Waring and Kenyon, 1990), we propose that the intronic enhancer permits transient expression of *pal-1* in a lineage (AB) in which it is normally repressed (Hunter and Kenyon, 1996). This is consistent with a previous report of transient V6 *pal-1* expression in hermaphrodite embryos (Edgar et al., 2001). Interpreted more widely, this specificity of *pal-1* expression to certain epidermal cell lineages but not others, suggests that the seam cells are not born equal; transcriptional differences between embryonic seam cells (leading to differences in cell behaviour), may contribute to differences in later, larval seam cell development.

Numerous downstream targets of PAL-1 have been identified in the C lineage (Baugh et al., 2005), and it is possible that these targets are also activated upon enhancer-driven expression of *pal-1* in the embryonic AB seam lineage. These include *vab-7*, *tbx-8* and *tbx-9*, which together form a regulatory unit that is required for, amongst other functions, correct embryonic epidermal cell positioning, including that of the seam cells (Pocock et al., 2004). Other potential targets of *pal-1* identified include the nuclear hormone receptors NHR-25, required for both embryonic elongation and the maintenance of cell-cell adherens junctions within the seam during larval development (Chen et al., 2004; Silhankova et al., 2005), and NHR-60, knockdown of which has also been shown to result in seam cell mis-positioning during embryogenesis (Simeckova et al., 2007). Further experiments would be required to assess whether the function of any of these genes within the epidermis is dependent on *pal-1*. Our finding that interference with *wrm-1* function also produces a seam overlap phenotype (albeit affecting a wider range of seam cells), together with the mutant phenotypes discussed above, suggest that V/P sorting roles may not be unique to *pal-1.* Wnt control of V/P sorting has not been demonstrated before, and a direct regulatory interaction with *pal-1* (presumably among other targets, given the wider range of cells affected by loss of *wrm-1* function) remains an intriguing possibility. An unbiased screen for mutants with mis-polarized seam cell adherens junction processes at L1 might yield additional important insights.

### 3.2 *pal-1/caudal* may have more widespread roles in tissue morphogenesis

We have detected a novel role for *pal-1*/*caudal* in regulating the embryonic movement of V3. In an original report of *pal-1* expression, it was remarked that *pal-1* appears to be expressed in particular cells that are involved in morphogenesis events and thus undergoing cell movement (Edgar et al., 2001). For example, it was described that the MS descendants mu intR and the mesoblast M, which normally complete substantial posterior migrations along the ventral midline during morphogenesis in wild-type embryos, did not migrate in *pal-1* null mutants. No phenotype for *pal-1* was observed in the E lineage, although the two *pal-1*-expressing cells are unusual in that they intercalate during formation of the gut. Thus, *pal-1*/*caudal* appears to have widespread roles in controlling cell movement and tissue morphogenesis.

Cdx1 and Cdx2, two of the three mammalian orthologues of *pal-1*/*caudal*, have been implicated in the formation of epithelial-derived tumours (Chawengsaksophak et al., 1997; Soubeyran et al., 2001; Domon-Dell et al., 2003; Salari et al., 2012; Hryniuk et al., 2014). Intriguingly, Cdx2 has also been described as a master regulator of metastasis (Mallo et al., 1998; Chun et al., 2007; Gross et al., 2008; Li et al., 2015), acting to promote cytoskeletal remodelling and formation of cell-cell junctions that inhibit cell migration (Lorentz et al., 1997; Keller et al., 2004; Platet et al., 2017). Thus, the data presented here, describing a role for *pal-1*/*caudal* in orchestrating epithelial cell movement in *C. elegans*, may be of interest in the context of understanding the role of Cdx genes in cancer progression.

### 3.3 Importance of embryonic cell positioning for the regulation of morphogenesis later in development

Our results suggest that during ventral epithelial morphogenesis the position of the seam daughter cell (with respect to junctions between the more ventral P cells) is crucial for determining ventral migration, regardless of cell fate, as we can manipulate the system (via the *pal-1e2091* allele, or loss of *lin-14* function) to put seam-fated daughters in positions normally occupied by hyp7-fated daughters, and they then migrate ventrally, branching the seam line.

This dependence of correct larval morphogenesis on earlier embryonic events specified by *pal-1* provides an interesting example of contingency during development. *pal-1-*dependent embryonic sorting (*i.e.* dorsal movement of V3 in embryos to enable its intercalation between V2 and V4) sets up the correct relative cell positioning and cell-cell contacts between V and P cells required during L1. Following L1 seam cell division, anterior daughters rely on their position, as opposed to their fate, to make the correct ventral movements in order to complete the ventral epidermis. Thus, cell position, leading to the correct polarity of the seam cell projections at L1, has already been established by the end of embryogenesis through *pal-1* mediated sorting of adjacent V and P cells.

### 3.4 Late completion of ventral epithelial morphogenesis during *C. elegans* development

It is curious that, in contrast to the dorsal epidermis, there is no dedicated ventral epidermis in the posterior of *C. elegans* larvae at hatching. Instead, an epidermal role is taken by the P cells. This is a transient role for the P cells, which go on to migrate to the ventral midline and differentiate into neuroblasts at the end of the first larval stage (Sulston and Horvitz, 1977). It is noteworthy that the ventral projections from the seam cells at this stage ensure that all anterior seam cell daughters migrate ventrally, where they are needed to complete the ventral portion of the hyp7 syncytium, whereas after this stage, migration of anterior seam daughters can take place in either the ventral or dorsal direction to increase the ploidy of hyp7.

This mode of development, in which extensive tissue re-arrangements occur post-hatching, contrasts with the development of many other nematodes, including *Pristionchus pacificus* and *Ascaris* parasites, which do not hatch until the second juvenile stage (equivalent to L2) or later, and thus complete the epidermal sheath inside the egg. In the case of *C. elegans*, therefore, it could be said that embryogenesis is completed *ex ovo*.

## 4 Experimental procedures

### 4.1 Nematode culture and genetics

All manipulations and maintenance of strains were performed as previously described (Wood, 1988). Strains used in this study were derived from the N2 Bristol wild-type, and are listed in the table below. SV1009 was a kind gift of Sander van den Heuvel (University of Utrecht).

**Table:** 

Strain name	Genotype
AW298	*wIs78 [ajm-1::gfp ​+ ​scm::gfp ​+ ​F58E10(cosmid)* ​+ ​*unc-119*^*+*^*]* IV; *him-5(e1490)* V
AW712	*pal-1(e2091)* III; *wIs78 [ajm-1::gfp ​+ ​scm::gfp ​+ ​F58E10(cosmid)* ​+ ​*unc-119* ​+ ​*]* IV; *him-5(e1490)* V
AW802	*pal-1(e2091) unc-119(ed3)* III; *ouEX674[pAW634(pal-1::gfp)* ​+ ​*unc-119* ​+ ​*]*
AW828	*pal-1(e2091)* III; *heIs63 [wrt-2p::gfp::PH ​+ ​wrt-2p::gfp::H2B ​+ ​lin-48p::mCherry]* V
AW1170	*pal-1(e2091) unc-119(ed3)* III; *him-5(e1490) heIs63[wrt-2p::gfp::PH ​+ ​wrt-2p::gfp::H2B ​+ ​lin-48p::mCherry]* V; *ouEx866[pAW634(pal-1::gfp)* ​+ ​*unc-119* ​+ ​*]*
AW1171	*pal-1(e2091) unc-119(ed3)* III; *him-5(e1490) heIs63[wrt-2p::gfp::PH ​+ ​wrt-2p::gfp::H2B ​+ ​lin-48p::mCherry]* V; *ouEx867[pAW857(pal-1A2110G::gfp)* ​+ ​*unc-119* ​+ ​*]*
AW1177	*heIs63 [wrt-2p::gfp::PH ​+ ​wrt-2p::gfp::H2B ​+ ​lin-48p::mCherry]* V*; lin-14(n179)ts* X
AW1448	*unc-119(ed3)* III; *ouEX996[pAW893(pal-1A2110Gintronx3::gfp)* ​+ ​*unc-119* ​+ ​*]*
AW1450	*unc-119(ed3)* III; *ouEX998[pAW892(pal-1WTintronx3::gfp)* ​+ ​*unc-119* ​+ ​*]*
OH3192	*ntIs1[gcy-5p::GFP ​+ ​lin-15* ​+ ​*]* V
SV1009	*heIs63[wrt-2p::gfp::PH ​+ ​wrt-2p::gfp::H2B ​+ ​lin-48p::mCherry]* V
JR667	*wIs51[scm::gfp ​+ ​unc-119* ​+ ​*]* V
EW95	*wrm-1(ne1982)ts* III; *wIs51[scm::gfp ​+ ​unc-119* ​+ ​*]* V
AW1172	*wrm-1(ne1982)ts* III; *heIs63 [wrt-2p::gfp::PH ​+ ​wrt-2p::gfp::H2B ​+ ​lin-48p::mCherry]* V

### 4.2 Cloning

All PCR reactions used for cloning were carried out using Phusion® High-Fidelity DNA Polymerase from New England Biolabs. All DNA constructs were verified by sequencing prior to use.

### 4.3 pAW634 *pal-1*::*gfp*

The upstream intergenic region and ORF up to but not including the stop codon of *pal-1* were amplified from genomic DNA using primers ACCAGCTATCTAACTATTCCCAACAG and CGACCTGCAGGCATGCAAGCtTAGCCGAATCTTCTGTTTGTCAC, the latter containing a complementary tag to the start of GFP from pPD95.75 (Fire lab vector). Simultaneously, GFP was amplified from pPD95.75 using primers GCTTGCATGCCTGCAGGTCG and AAGGGCCCG TACGGCCGACTAGTAGG. The products were then fused in a single PCR reaction using primers ACCAGCTATCTAACTATTCCCAACAG and AAACAGTTATGTTTGGTATATTGGG, before being T/A cloned into a pCR®-XL-TOPO® plasmid (Invitrogen) to make pAW634.

### 4.4 pAW857 *pal-1(A2110G)*::*gfp*

A region spanning the 5th and 6th exons of *pal-1(e2091)* was PCR amplified using primers CGACCTGCAGGCATGCAAGCTTAGCCGAATCTTCTGTTTGTCAC (includes a redundant overlap for GFP tagging) and gtaagttttcaaaagagtctccccg from genomic DNA and T/A cloned into the pCR®-XL-TOPO® plasmid. This plasmid was subjected to site-directed mutagenesis of the 3’ mutation in the intron back to wild-type using overlapping mismatched primers ctacagaaatgataaagcaaggGtttctgaccagctcgcagag and ctctgcgagctggtcagaaaCccttgctttatcatttctgtag. The *pal-1* 5th intron containing only A2110G was then sub-cloned into a pCR®-XL-TOPO® backbone using TCTAGAGTCGACCTGCAGGCATGC AAGC and cccaattgtcgacgatgttaagcttgac. This was then cloned back into the full-length *pal-1*::*gfp* reporter construct using SalI to create pAW857.

### 4.5 pAW892 and pAW893

**Triple tandem repeat of the *pal-1* wild-type and A2110G intronic enhancers into pPD107.94** The triple tandem repeat of the *pal-1* A2110G intronic enhancer region of the last intron was generated using PCR. Primers with homologous ends (ggtggcaaattgggatACGATGTTAAGCTTGAC AGAACGAC and GTCAAGCTTAACATCGTatcccaatttgccacctctcatc) generated a ‘ladder’ of PCR products after amplification due to the annealed products of the primers. The ‘rung’ that corresponded to a triple repeat of the enhancer region was gel purified and cloned into pCR®-XL-TOPO®, before cloning into pPD107.94 (Fire lab vector) using XbaI and SpeI into XbaI non-directionally to make pAW892 (wild-type) and pAW893 (A2110G).

### 4.6 Generation of transgenic lines

Transgenic worm lines were generated as previously described (Mello et al., 1991; Mello and Fire, 1995). All constructs were injected (at a concentration of 10–20 ​ng/μl) into an unc-119(ed3) background using a rescuing unc-119 ​+ ​plasmid (pDP#MM016β) as a co-injection marker (Maduro and Pilgrim, 1995). Lines were likewise maintained by selection for the wild-type *unc-119* phenotype. Several transgenic lines were generated and analysed for each construct investigated.

### 4.7 Microscopy and live imaging

An epifluorescent Zeiss microscope fitted with Nomarski (DIC) and GFP filters and a 63x oil immersion objective and Axiovision software was used to capture fluorescent and DIC images. Worms were mounted on 2% agarose pads containing 0.5% phenoxypropanol in order to immobilize them. A Leica SP5 laser scanning confocal fitted with a 488 ​nm argon laser was used to capture some fluorescent images of larval seam cells. An Ultra-VIEW VoX PerkinElmer spinning disk confocal mounted on a IX81 Olympus microscope fitted with a 60 ​× ​1.3 NA silicon immersion objective, a 488 ​nm laser line and Volocity software was used to capture images of live *ajm-1*::*gfp* embryos. Very high laser power (80%) was used to minimize z stack acquisition time in order to obtain static images of twitching embryos.

### 4.8 Lineaging

Embryonic lineaging was performed as described in (Schnabel et al., 1997), using the lineage analysis software SIMI°BioCell. For each full lineage, the 6 ​V cells and 6 ​P cells from one side of the embryo (left or right) were tracked from the one-cell stage until the 1.5 fold stage allowing a 4D-reconstruction of cell positions and migrations. Wild-type lineages were obtained from two embryos; one lineage from strain OH3192 and two from AW298. Images shown are data from AW298. *pal-1(e2091)* lineages were obtained from two embryos; two from AW828 and one from a non-array carrying animal from strain AW802. Images shown are data from AW802. *pal-1(e2091)* rescue and expression lineages were obtained from four lineages from three AW802 animals carrying the rescuing transgenic array. All express GFP in the pairs of sister cells, V3/P7 and V4/V6, and rescue the *e2091* V3/V4 seam cell a-p mis-ordering.

## Author contributions

S. G., T. M., R. P., and A. W. designed the experiments, S. G., T. M. and R. P. conducted the experiments and S. G. and A. W. wrote the paper with contribution from R. P. and T. M.

## Declaration of competing interest

The authors declare they have no competing interests.

## Appendix A Supplementary data

Supplementary Fig. 1 Stages of P cell migration. Schematic of P cell migration on the ventral surface of the worm during L1. P cells are shown in yellow, P cell nuclei in brown, hyp7 in grey and seam cells in green. At hatching, P cells make up a large portion of the ventral epidermis. During L1, P cell nuclei migrate to the ventral midline, followed by the shrinking and migration of the P cell body. Upon reaching the ventral midline, the opposing pairs of P cells intercalate with each other and form up in a single line along the ventral midline of the worm. Supplementary Fig. 1

Supplementary Fig. 2 Alignment of the 5th intron of *pal-1* between orthologues in *C. elegans*, *C. briggsae* and *C. remanei*. Black boxed areas indicate the flanking exons. Regions of high conservation are indicated in red; low conservation is shown in blue. Both mutated bases of the *e2091* allele, indicated with asterisks, are conserved, but whereas the 5′ most base change (A2110G) is within a highly conserved sequence motif, the 3′ most base change (G3004A) is not. The region in which a potential TCF binding site was identified, that would be mutated in the *e2091* allele, is indicated with a green line. Supplementary Fig. 2

Supplementary Fig. 3 *wrm-1(ne1982)ts* superficially phenocopies the *pal-1(e2091)* seam cell overlap phenotype. A. A *wrm-1(ne1982)ts* animal that has been temperature shifted to a restrictive temperature of 26.5°C partway through embryogenesis (gravid hermaphrodites, grown at 15°C, were bleached and eggs left on plates at 20 ​°C for 3 ​h before being moved to the restrictive temperature of either 25 ​°C or 26.5 ​°C; animals were scored upon hatching). The seam cells exhibit a similar overlapped phenotype that can be observed in *pal-1(e2091)* animals, although the overlap affects a wider range of cells and can happen twice in the same seam line (never observed in *pal-1(e2091)* animals). Scale bar ​= ​10 ​μm. B. Percentage of newly hatched animals that exhibit a seam overlap phenotype at each restrictive temperature. Over 30% of animals are affected at 26.5°C. (47 ​≤ ​n ​≤ ​81 per data set, Student’s T-Test: * ​= ​P ​≤ ​0.01, *** ​= ​P ​≤ ​0.0001). Animals were shifted to both 25°C and 26.5°C due to previous findings that in order to perturb fate during post-embryonic divisions, worms containing the *wrm-1(ne1982)ts* allele had to be grown at 26.5°C, as 25°C was unable to cause sufficient loss of *wrm-1* function (Gleason and Eisenmann, 2010). C. Number of *scm*::*gfp*-positive nuclei after the L1 seam cell division at the restrictive temperatures. *scm*::*gfp* is an established marker of seam cell fate, and therefore can be used to determine whether the misplaced seam cells still retain their fate. After the L1 seam division, wild-type worms have 10 seam cells. This is unchanged in *wrm-1(ne1982)ts* mutants at 20°C and 26.5°C showing that the misplaced seam cells do indeed retain the seam cell fate, and are simply mis-positioned. At 25°C, the difference in seam cell number is significant (Student’s T-Test: * ​= ​P ​≤ ​0.01) although this difference does not persist or increase at 26.5°C and is not therefore likely to be coupled to the increase in seam cell overlap. (46 ​≤ ​n ​≤ ​58 per data set, error bars ​= ​S.E.M.). Supplementary Fig. 3
