# Supplementary material for: Caudal-dependent cell positioning directs morphogenesis of the C. elegans ventral epidermis
Source: Dev Biol. 2020 May 1;461(1):31–42. doi: 10.1016/j.ydbio.2020.01.001 (PMC7181193; doi:10.1016/j.ydbio.2020.01.001)
Supplement: Supplementary Fig. 1 — Stages of P cell migration. Schematic of P cell migration on the ventral surface of the worm during L1. P cells are shown in yellow, P cell nuclei in brown, hyp7 in grey and seam cells in green. At hatching, P cells make up a large portion of the ventral epidermis. During L1, P cell nuclei migrate to the ventral midline, followed by the shrinking and migration of the P cell body. Upon reaching the ventral midline, the opposing pairs of P cells intercalate with each other and form up in a single line along the ventral midline of the worm. [file mmc4.pdf]

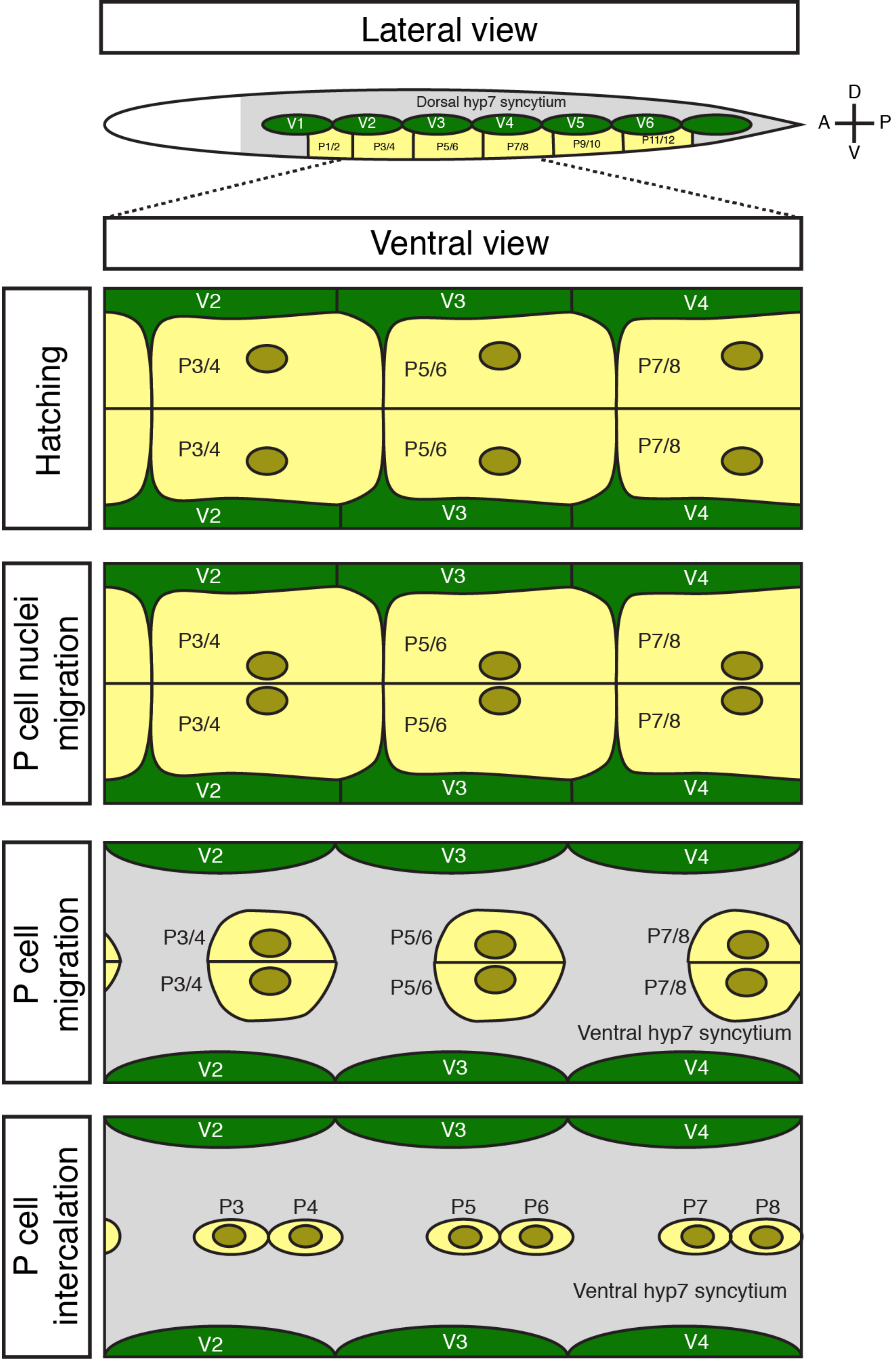

Lateral view
Dorsal hyp7 syncytium
V1
V2
V3
V4
V5
V6
P1/2
P3/4
P5/6
P7/8
P9/10
P11/12
D
A
P
V
Ventral view
Hatching
P cell nuclei migration
P cell migration
Ventral hyp7 syncytium
P cell intercalation
P3
P4
P5
P6
P7
P8
