# Supplementary material for: Caudal-dependent cell positioning directs morphogenesis of the C. elegans ventral epidermis
Source: Dev Biol. 2020 May 1;461(1):31–42. doi: 10.1016/j.ydbio.2020.01.001 (PMC7181193; doi:10.1016/j.ydbio.2020.01.001)
Supplement: Supplementary Fig. 2 — Alignment of the 5th intron of pal-1 between orthologues in C. elegans, C. briggsae and C. remanei. Black boxed areas indicate the flanking exons. Regions of high conservation are indicated in red; low conservation is shown in blue. Both mutated bases of the e2091 allele, indicated with asterisks, are conserved, but whereas the 5′ most base change (A2110G) is within a highly conserved sequence motif, the 3′ most base change (G3004A) is not. The region in which a potential TCF binding site was identified, that would be mutated in the e2091 allele, is indicated with a green line. [file mmc5.pdf]

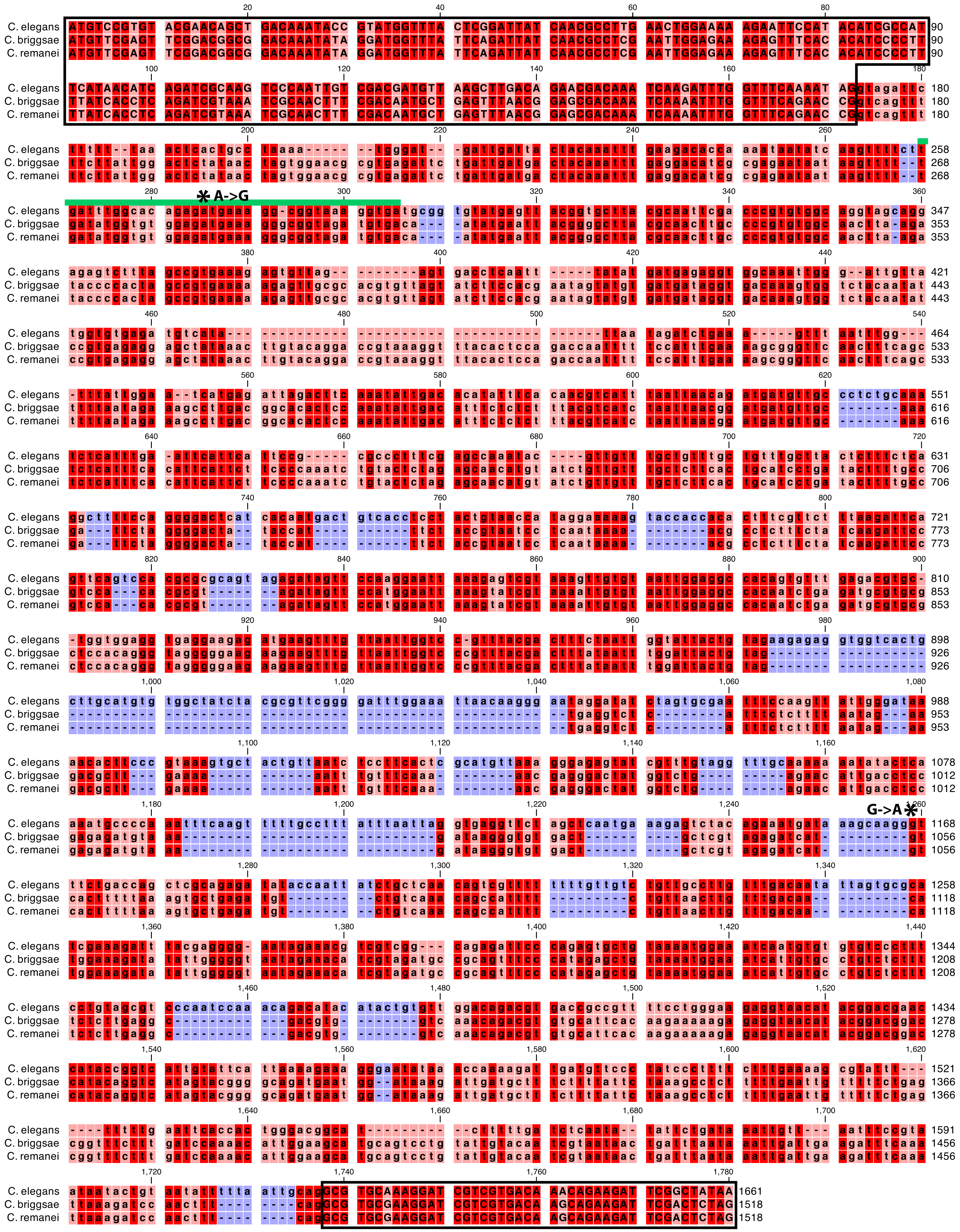

C. elegans
C. briggsae
C. remanei
A->G
G->A
