# Supplementary figures and images for: Caudal-dependent cell positioning directs morphogenesis of the C. elegans ventral epidermis
Source: Dev Biol. 2020 May 1;461(1):31–42. doi: 10.1016/j.ydbio.2020.01.001 (PMC7181193; doi:10.1016/j.ydbio.2020.01.001)

**A**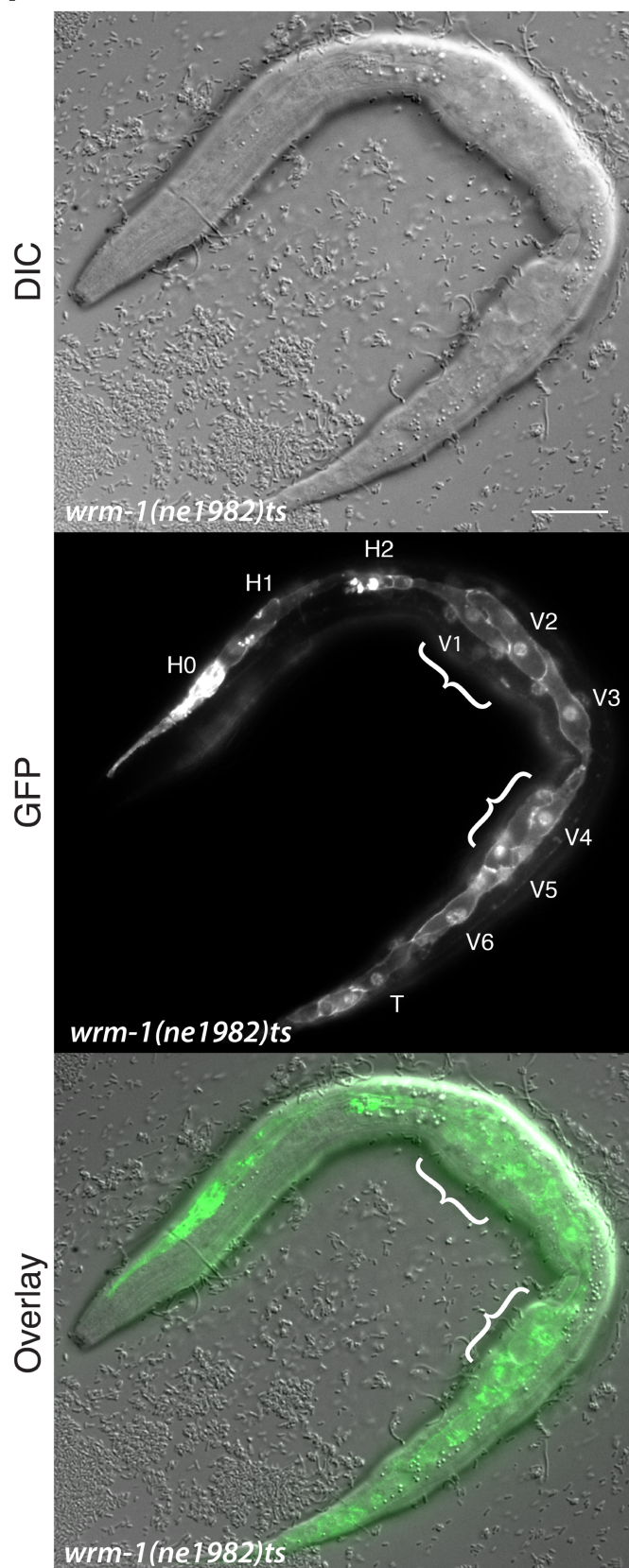**B**

% L1s with seam cell overlap

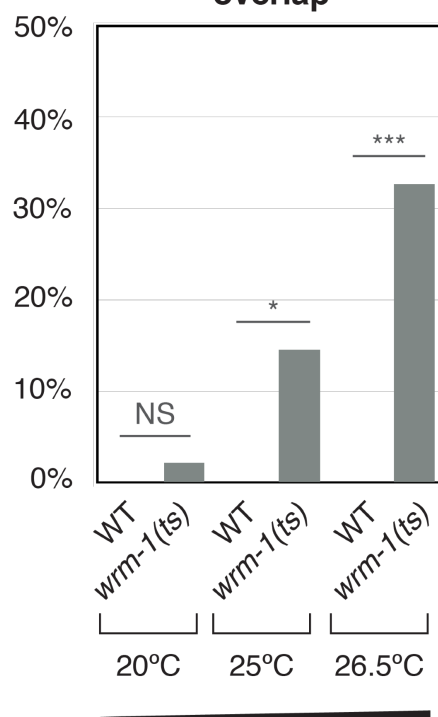**C**

No. of *scm::gfp*-positive nuclei in late L1

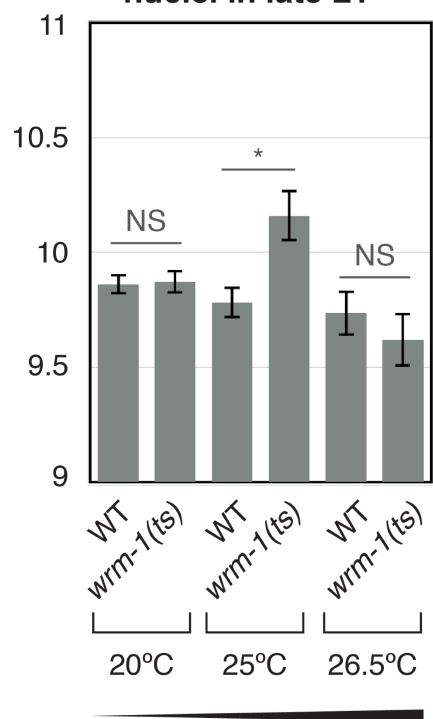

Supplement: Supplementary Fig. 3 — wrm-1(ne1982)ts superficially phenocopies the pal-1(e2091) seam cell overlap phenotype. A. A wrm-1(ne1982)ts animal that has been temperature shifted to a restrictive temperature of 26.5°C partway through embryogenesis (gravid hermaphrodites, grown at 15°C, were bleached and eggs left on plates at 20 ​°C for 3 ​h before being moved to the restrictive temperature of either 25 ​°C or 26.5 ​°C; animals were scored upon hatching). The seam cells exhibit a similar overlapped phenotype that can be observed in pal-1(e2091) animals, although the overlap affects a wider range of cells and can happen twice in the same seam line (never observed in pal-1(e2091) animals). Scale bar ​= ​10 ​μm. B. Percentage of newly hatched animals that exhibit a seam overlap phenotype at each restrictive temperature. Over 30% of animals are affected at 26.5°C. (47 ​≤ ​n ​≤ ​81 per data set, Student’s T-Test: * ​= ​P ​≤ ​0.01, *** ​= ​P ​≤ ​0.0001). Animals were shifted to both 25°C and 26.5°C due to previous findings that in order to perturb fate during post-embryonic divisions, worms containing the wrm-1(ne1982)ts allele had to be grown at 26.5°C, as 25°C was unable to cause sufficient loss of wrm-1 function (Gleason and Eisenmann, 2010). C. Number of scm::gfp-positive nuclei after the L1 seam cell division at the restrictive temperatures. scm::gfp is an established marker of seam cell fate, and therefore can be used to determine whether the misplaced seam cells still retain their fate. After the L1 seam division, wild-type worms have 10 seam cells. This is unchanged in wrm-1(ne1982)ts mutants at 20°C and 26.5°C showing that the misplaced seam cells do indeed retain the seam cell fate, and are simply mis-positioned. At 25°C, the difference in seam cell number is significant (Student’s T-Test: * ​= ​P ​≤ ​0.01) although this difference does not persist or increase at 26.5°C and is not therefore likely to be coupled to the increase in seam cell overlap. (46 ​≤ ​n ​≤ ​58 per data set, error bars [file mmc6.pdf]
